# SMARCA4-deficient carcinoma of the head and neck region: report of 8 new sinonasal and non-sinonasal cases and literature review

**DOI:** 10.1007/s00428-026-04459-5

**Published:** 2026-03-09

**Authors:** Mihaela Farkas, Abbas Agaimy, Marián Švajdler, Lukáš Hauer, Petr Martínek, Tomáš Vaněček, Kristýna Pivovarčíková, Barbora Prchlíková, Roderick H. W. Simpson, Klaudia Gočárová, Peter Vereš, Petr Slavík, Kristýna Behenská, Ksenija Marjanovic, Michal Michal, Alena Skálová, Martina Bradová

**Affiliations:** 1 Onco Team Diagnostic, Bucharest, Romania; 2 Comprehensive Cancer Center (CCC) Erlangen-EMN, Institute of Pathology, University Hospital Erlangen, Erlangen, Germany; 3 Department of Pathology, Faculty of Medicine in Plzen, Charles University, University Hospital Plzen, Dr. E. Benese 13, 305 99 Plzen, Czech Republic; 4 Bioptic Laboratory, Ltd, Plzen, Czech Republic; 5 Cytopathos, Ltd, Bratislava, Slovakia; 6 Department of Oral and Maxillofacial Surgery, Faculty of Medicine in Plzen, University Hospital and Faculty of Medicine in Pilsen, Charles University, Plzen, Czech Republic; 7 Department of Pathological Anatomy, Rudolf and Stefanie Hospital Benešov, Inc., Benešov, Czech Republic; 8 Department of Pathology, University of Calgary, Calgary, AB Canada; 9 Faculty of Medicine, St. Elizabeth Cancer Institute, Ltd., 1 st Oncology Clinic, Comenius University in Bratislava, Bratislava, Slovakia; 10 Medicyt, s.r.o., Bratislava, Slovakia; 11 Faculty of Medicine Osijek, Josip Juraj Strossmayer University of Osijek, Osijek, Croatia; 12 Department of Pathology and Forensic Medicine, Osijek University HospitalOsijek, Croatia

**Keywords:** Oral cavity, SMARCA4-deficient carcinoma, Non-sinonasal, Sinonasal

## Abstract

Neoplasms with inactivating mutations in SWI/SNF chromatin remodeling complex subunits gained attention in the head and neck (H&N) region due to their poor clinical outcomes. The sinonasal tract is a recognized “hot-spot” for SMARCA4-deficient cancers, including SMARCA4-deficient sinonasal carcinomas, and most sinonasal teratocarcinosarcomas. To date, only two H&N SMARCA4-deficient carcinomas have been reported outside the sinonasal region. We identified eight cases of SMARCA4-deficient H&N carcinomas from the authors’ files and reviewed their clinicopathological features. All cases with available tissue blocks were investigated by molecular genetic methods using next-generation sequencing (NGS). The cohort included four sinonasal and four non-sinonasal tumors involving the tongue, oral floor, the upper jaw submucosa, and the hypopharynx. The extra-sinonasal cases affected three males and one female (ages 61–81). Histologically, two cases showed poorly differentiated small cell morphology, one case resembled salivary duct carcinoma, and one case had a dedifferentiated squamous cell carcinoma phenotype. Regional lymph node metastases were documented in two cases. Sinonasal tumors showed spindle cell morphology, olfactory neuroblastoma-like features with glandular or squamoid differentiation areas, and an undifferentiated small cell pattern. This study expands the known anatomical distribution and histological spectrum of SMARCA4-deficient H&N carcinomas. Their occurrence outside the sinonasal tract is rare and poses a diagnostic challenge. These tumors are highly aggressive and often present at advanced stages. In the sinonasal region, they can mimic olfactory neuroblastoma, while in other H&N sites, they can resemble neuroendocrine carcinoma.

**Supplementary Information:**

The online version contains supplementary material available at 10.1007/s00428-026-04459-5.

## Introduction

SMARCA4-deficient neoplasms are rare and highly aggressive malignancies characterized by inactivation or loss of the *SMARCA4* gene, which encodes BRG1, a catalytic subunit of the SWI/SNF chromatin remodeling complex [1–3]. These neoplasms exhibit a pattern-based association with specific cancer types rather than a random cancer distribution [3]. Initially, SMARCA4-homozygous inactivation or haploinsufficiency was described in a small subset of malignant rhabdoid tumors and in the majority of small cell carcinoma of the ovary, hypercalcemic type (SCCOHT), including its large cell variant [4–7]. With the advent of advanced genetic testing, the spectrum of SMARCA4-deficient neoplasms has expanded and now includes a subset of non-small cell lung carcinomas [8, 9], rare cases of epithelioid sarcoma of the proximal type [10], thoracic SMARCA4-deficient undifferentiated tumor [11], a subset of malignant rhabdoid tumors [12], and a variety of dedifferentiated and undifferentiated carcinomas and sarcomas involving endometrium [13, 14], digestive tract [15, 16], the pancreas, and urogenital system [17–20]. In the head and neck, SMARCA4-deficient malignancies include SMARCA4-deficient sinonasal carcinoma and the vast majority of sinonasal teratocarcinosarcomas [21].

SMARCA4-deficient sinonasal carcinomas are high-grade anaplastic tumors that frequently show necrosis and consist of either large epithelioid or rhabdoid cells or show small-cell morphology with abortive pseudorosettes, mimicking olfactory neuroblastoma (ONB), while lacking squamous or glandular features [22–24]. In addition to loss of SMARCA4 (BRG1) expression, these tumors show consistent immunoreactivity for pankeratins (with or without CK7 expression), variable positivity for neuroendocrine markers and absence of CK5, p63, p40, or p16 [23].

Within the H&N region, SMARCA4-deficient carcinoma is predominantly confined to the sinonasal tract; involvement of other sites is extraordinary, with only two cases reported in the literature [25, 26]. Herein, we summarize our experience with head and neck SMARCA4-deficient carcinomas and present four sinonasal and four non-sinonasal cases, widening the topographic distribution of these lesions. A brief review of the literature is also provided.

## Materials and methods

### Case selection

The study was conducted in accordance with the guidelines of the Ethics Committee of the Faculty Hospital in Pilsen. Informed consent was not required due to the retrospective nature of the analysis. Eight cases of SMARCA4-deficient carcinomas of the head and neck were identified from the authors’ institutional archives. The diagnosis in each case was confirmed by immunohistochemistry demonstrating a loss of SMARCA4 protein expression. Follow-up data were collected from referring pathologists, treating clinicians, and hospital medical records.

### Histology and immunohistochemistry

For conventional microscopy, the excised tissues were fixed in formalin, processed routinely, embedded in paraffin (FFPE), cut, and stained with hematoxylin and eosin.

For immunohistochemistry, 4-μm-thick sections were cut from paraffin blocks and mounted on positively charged slides (TOMO, Matsunami Glass IND, Osaka, Japan). Sections were processed on a BenchMark ULTRA (Ventana Medical Systems, Tucson, AZ), deparaffinized, and subjected to heat-induced epitope retrieval by immersion in a CC1 solution (pH 8.6) at 95 °C and CC2 solution (pH 6.0) at 92 °C. All primary antibodies used in this study are summarized in Table 1.

**Table 1:** Antibodies used for immunohistochemical study

Antibody specificity	Clone	Dilution	Antigen retrieval/time	Source
SMARCA4	EPNCIR111A	1:1000	CC1/52 min	Abcam
SMARCB1	MRQ-27	RTU	CC1/52 min	Ventana
SMARCA2	Polyclonal	1:200	CC2/56 min	Atlas Antibodies AB
AE1/3	AE1/AE3 + PCK26	RTU	CC1/20 min	Ventana
OSCAR	IsoType:IgG2a	1:100	EnVision High pH/30 min	Covance
CAM5.2	CAM5.2	RTU	CC1/36 min	Ventana
Claudin 4	3E2C1	1:200	CC1/52 min	Thermo Scientific–Life Technologies
MOC-31	MOC-31	1:50	CC1/52 min	Abcam
CK5/6	D5/16B4	1:50	EnVision High pH/30 min	Dako
CK7	OV-TL 12/30	RTU	EnVision High pH/30 min	Dako
p63	DAK-p63	RTU	EnVision Low pH/30 min	Dako
p40	BC28	RTU	CC1/52 min	Biocare Medical
Synaptophysin	SP11	RTU	CC1/52 min	Dako
Chromogranin A	DAK-A3	1:100	CC1/20 min	Dako Cytomation
CD56	123C3	RTU	CC1/64 min	Ventana
INSM1	A-8	1:1000	CC1/64 min	Santa Cruz
p16	R15A	1:100	EnVision FLEX TRS High pH/30min	DB Biotech
NUT	C52B1	1:100	CC1/64 min	Cell Signaling
SOX 10	SP267	RTU	CC1/64 min	Cell Marque
S-100 protein	Polyclonal	RTU	EnVision High pH/30 min	Dako
p53	DO-7	RTU	EnVision High pH/30 min	Ventana
Ki-67	MIB-1	RTU	EnVision High pH/30 min	Dako
MyoD1	EP212	RTU	CC2/68	Ventana
Desmin	D33	1:200	EnVision High pH/30 min	Dako

*RTU* ready to use, *CC1* EDTA buffer pH 8.6 at 95 °C, *CC2* citrate buffer pH 6.0 at 92 °C; *min* minutes; EnVision High pH 9.0 at 97 °C; EnVision Low pH 6.0 at 97 °C

Visualization was performed using the ultraView Universal DAB Detection Kit (Roche, Tucson, AZ) or the ultraView Universal Alkaline Phosphatase Red Detection Kit (Roche, Tucson, AZ). The slides were counterstained with Mayer’s hematoxylin. Appropriate positive and negative controls were employed.

### Review of the literature

PubMed database was searched for relevant peer-reviewed reports published in English, using a combination of the following keywords: “SMARCA4,” “BRG1,” “SWI/SNF,” “deficient carcinoma,” and/or “head and neck.” From over 3000 articles published in the period from 1992 to December 2024, we selected all relevant studies dealing with SMARCA4/BRG1-deficient carcinomas of the head and neck. Eight articles with detailed histological and immunohistochemical descriptions were identified, and the clinicopathological information was extracted and analyzed together with our data.

#### Genetic analysis using next-generation sequencing

### Cases 1, 2, 5, 7, and 8

## Illumina TruSight Oncology 500 and TruSight RNA Pan-Cancer Panel assays

All cases were analyzed using the TruSight Oncology 500 assay from Illumina. This panel analyzes both DNA and RNA. The DNA analysis interrogates 523 genes for single nucleotide variants (SNVs) and indels. For the RNA analysis, the original set of probes for fusion detection (which interrogates only 55 genes) was replaced with the TruSight RNA Pan-Cancer Panel (Illumina) targeting 1385 genes. The complete list of genes in both the DNA and RNA parts of the analysis can be found on the manufacturer’s website [27, 28]. Data analysis for fusion detection was performed using the DRAGEN RNA app version 4.0.4 (Illumina). The analysis was otherwise performed as reported previously [29].

## Copy number variant detection using low-pass whole genome sequencing

For low-pass whole genome sequencing, up to 3 FFPE sections (10 µm thick) were used and DNA was extracted using Maxwell RSC DNA FFPE Kit on Maxwell RSC 48 Instrument (Promega, Madison, WI, USA). DNA was quantified using the Qubit Broad Range DNA Assay (Thermo Fisher Scientific).

DNA fragmentation and library construction were performed following the Kapa EvoPlus V2 Library kit (Roche Diagnostics, Mannheim, Germany). Dual indexed libraries were sequenced on a Novaseq 6000 (Illumina, San Diego, CA) to achieve at least 10 M reads per sample. The analysis of the sequencing results was performed using FREEC software [30].

### Cases 3 and 4

DNA extraction method was performed as reported previously [31]. Customized DNA NGS panel encompassing 30 cancer-related genes was used (*AKT1*, *ALK*, *BRAF*, *CTNNB1,EGFR*, *FGFR1*, *FGFR2*, *FGFR3*, *FGFR4*, *GNA11*, *GNAQ*, *HRAS*, *IDH1*, *IDH2*, *KEAP1*, *KIT*, *KRAS*, *MAP2K1 MET*, *NRAS*, *NTRK1*, *NTRK2*, *NTRK3*, *PDGFRA*, *ERBB2*, *PIK3CA*, *PTEN*, *RET*, *ROS1*, *STK11*, *TP53*, and *TRDMT1*).

## Detection of loss SMARCA4 gene by FISH (all cases except Case 6)

Prior to performing FISH, hematoxylin and eosin–stained slides were examined to determine the areas for cell counting. Then, a 4-µm-thick formalin-fixed, paraffin-embedded section was placed onto a positively charged slide. The unstained slide was routinely deparaffinized and incubated in the 1 × Target Retrieval Solution Citrate pH 6 (Dako, Glostrup, Denmark) for 40 min at 95 °C, subsequently cooled for 20 min at room temperature in the same solution and washed in deionized water for 5 min. The slide was digested in protease solution with pepsin (0.5 mg/mL) (Sigma-Aldrich, St Louis, MO, USA) in 0.01 M HCl at 37 °C from 45 to 60 min according to the sample conditions. The slide was then rinsed in deionized water for 5 min, dehydrated in a series of ethanol solutions (70%, 85%, 96% for 2 min each), and air-dried.

For the detection of the deletion of the *SMARCA4* gene, we used our own designed SureFish SMARCA4 (19p13.2) specific probe and FOSB (19q13.32) control probe (both Agilent Technologies, Santa Clara, CA, USA). Chromosomal regions for the *SMARCA4* probe were chr19: 11070968–11173383 and for the *FOSB* probe were chr19: 45978437–46378436. Both probes were mixed with water and LSI/WCP hybridization buffer (Vysis/Abbott Molecular, IL, USA) in a 1:1:1:7 ratio, respectively.

An appropriate amount of mixed probe was applied on the specimens, covered with a glass cover slip, and sealed with rubber cement. The slide was incubated in the ThermoBrite instrument (StatSpin/Iris Sample Processing, Westwood, MA, USA) with codenaturation parameters of 85 °C for 8 min and hybridization parameters of 37 °C for 16 h. The rubber-cemented cover slip was then removed, and the slide was placed in posthybridization wash solution (2 × SSC/0.3% NP-40) at 72 °C for 2 min. The slides were air-dried in the dark, counterstained with DAPI II (Vysis/Abbott Molecular), covered with slip, and immediately examined under an Olympus BX51 fluorescence microscope using a ×100 objective and filter sets triple band pass (DAPI/Spectrum Green/Spectrum Orange), dual band pass (FITC/Spectrum Orange), and single band pass (Spectrum Green or Spectrum Orange).

Signals of *SMARCA4* and corresponding (control) signals of *FOSB* were counted in one hundred randomly selected nonoverlapping tumor cell nuclei. The samples were considered positive for heterozygous or homozygous deletion of *SMARCA4* if nuclei showed a positive pattern in ≥ 45% and ≥ 30%, respectively (cut-off was set as mean value in normal non-neoplastic control tissues + 3 standard deviations).

### Results

Eight cases of head and neck SMARCA4-deficient carcinoma were identified, and their clinicopathological features were summarized alongside previously reported cases (Tables 2 and 3, Supplementary file 1).

**Table 2:** Immunohistochemical findings of the 30 SMARCA4-deficient H&N carcinomas

Study	Case	SMARCA4	SMARCB1	SMARCA2/ARID1A	CK	CK7/CK5/6	syn/CHgA/CD56/INSM1	p63
**Current study Bradová et al.**	**Case 1**	Loss	Retained in both components	Loss in poorly dif. component/retained	Weak OSCAR, CAM5.2, AE1/3, MOC31 (neg Claudin 4)	neg in both/positive in SCC	W +/neg/ND/neg	neg
	**Case 2**	Loss	Retained	Loss/retained	OSCAR, AE1/3, CAM5.2 (neg Claudin 4)	neg/ND	W +/neg/ND/neg	neg
	**Case 3**	Loss	Retained	ND/retained	AE1/3 (Claudin 4 ND)	+/neg	ND/ND/ND/ND	neg
	**Case 4**	Loss	Retained	ND/retained	AE1/3 (Claudin 4 ND)	+/ND	ND/ND/ND/ND	neg
	**Case 5**	Loss	ND	ND/ND	F +,(neg Claudin 4)	ND	ND/ND/ND/ND	ND
	**Case 6**	Loss	ND	ND/ND	ND (Claudin 4 ND)	F +/ND	ND/ND/ND/ND	ND
	**Case 7**	Loss	Retained	ND/ND	AE1/3, OSCAR, CAM5.2; Claudin 4 +	ND/neg	+/neg/ND/F + (10%)	neg
	**Case 8**	Loss	Retained	Loss/retained	AE1/3, Claudin 4 +	neg/neg	+/W +/+/+	neg
**2017 Jo, et al.**	**Case 8**	Loss	Retained	ND/ND	+	ND/ND	+/neg/ND/ND	ND
**2020 Agaimy, et al.**	**Case 1**	Loss	Retained	Reduced/retained	AE1/3,	neg/neg	W +/neg/W +/ND	neg
	**Case 2**	Loss	Retained	Reduced/ND	+	neg/neg	+ + +/neg/F +/ND	neg
	**Case 3**	Loss	Retained	Loss/ND	+	neg/neg	W +/neg/F +/NF	neg
	**Case 4**	Loss	Retained	Retained/ND	+	F +/neg	+ +/neg/ND/ND	neg
	**Case 5**	Loss	Retained	Retained/ND	+	ND/neg	F +/F +/ND/ND	neg
	**Case 6**	Loss	Retained	Retained/ND	+	ND/neg	F +/F +/ND/ND	neg
	**Case 7**	Loss	Retained	Retained/ND	+	ND/neg	F +/F +/ND/ND	neg
	**Case 8**	Loss	Retained	Retained/ND	+	neg/neg	neg/neg/ND/ND	neg
	**Case 9**	Loss	Retained	ND/ND	+	ND/neg	W +/neg/ND/ND	neg
	**Case 10**	Loss	Retained	ND/ND	+	F +/neg	i +/neg/neg/ND	neg
2021 Kakkar, et al.*	**Case 1**	Loss	ND	Retained/ND	+	ND/ND	F +/F +/F +/F +	neg
	**Case 2**	Loss	ND	Retained/ND	+	neg/ND	+/+/neg/neg	neg
	**Case 6**	Loss	ND	Retained/ND	+	neg/neg	F +/F +/ND/F +	neg
	**Case 7**	Loss	ND	Retained/ND	+	In squamous foci only/ND	+/+/F +/neg	+ in squamous areas
**2021 Mibayashi, et al.**	**Case**	Loss	F loss	F loss/ND	F +	ND/ND	F +/F +/ND/ND	neg
**2023 Pasricha, et al.**	**Case**	Loss	Retained	ND/ND	+	neg/ND	neg/ND/ND/ND	neg
**2024 H Kang, et al.**	**Case 1**	Loss	ND	ND/ND	ND	ND/ND	ND/ND/ND/ND	ND
	**Case2**	Loss	ND	ND/ND	ND	ND/ND	ND/ND/ND/ND	ND
**2024 Zhu et al.**	**Case 1**	Loss	ND	ND/ND	ND	ND/ND	ND/ND/ND/ND	ND
	**Case 2**	Loss	ND	ND/ND	ND	ND/ND	ND/ND/ND/ND	ND
**2024 Thiagarajan, et al.**	**Case**	Loss	Retained	ND/ND	+	pos/ND	neg/ND/ND/neg	neg

^*^Cases 3–5 were previously reported by Agaimy et al. PMID: 31934917

+, positive; F, focal; ND, not done; neg, not done; syn, synaptophysin; CHgA, chromogranin A

**Table 3:** The molecular genetic results of SMARCA4-deficient sinonasal and non-sinonasal head and neck carcinomas

Case	TruSight RNA Pan-Cancer Panel	TruSight Oncology 500 Panel - DNA part	Kapa EvoPlus v2 and low-pass whole-genome sequencing	FISH SMARCA4 loss
Case 1	SLC66A1::PLCB1, exon 1::exon 31, frame-unknown, NM_001040125.2, NM_015192.4, chr1:19,639,383, chr20:8,770,822, Hg19	PRDM1 c.2067C > A, p.Cys(689Ter), (alias C689Ter), AF:52%, NM_001198.4, chr6:106,554,950, hg19 EP300 c.2683del, p.(Gln895AsnfsTer51), (alias Q895NfsTer51), AF:64%, NM_001429.4 chr22:41,546,064, hg19 SMARCA4 c.579_580delinsTT, p.(Gln194Ter), (alias Q194Ter), AF:52%, NM_003072.5, chr19:11,097,088, hg19 Clinically significant gene amplifications: CCND1 (19 ×), FGF19 (22 ×), FGF4 (20 ×), FGF3 (11 ×), KRAS (6.6 ×) LOH of chromosome 19p: Positive TMB: Low; 6.3 mut/Mb MSI-L; 3% loci	Multiple CNVs, probable loss on chromosome 19p	Positive (heterozygous)
Case 2	RPS6KB1::VMP1, exon 4::exon 6, in-frame, NM_003161.4, NM_030938.5, chr17:57,992,064, chr17:57,842,332, Hg19	TP53 c.592G > T, p.(Glu198Ter), (alias E198Ter), AF:70%, NM_000546.6, chr17:7,578,257, hg19 CDKN2A c.25_31dup, p.(Pro11HisfsTer6), (alias P11HfsTer6), AF:55%, NM_058195.4, chr9:21,974,795, hg19 Clinically significant gene amplifications: Negative LOH of chromosome 19p: Positive TMB: High; 22 mut/Mb MSI-L; 2% loci	Multiple CNVs, probable loss on chromosome 19p	Positive (homozygous)
Case 3	Negative	*KRAS* (c.35G > C, p.Gly12Ala): VAF:39%	***ND***	Positive (heterozygous) (note: susp. complete loss of chr. 19 due to loss of control probe on 19q13.32)
Case 4	ND	ND	ND	Positive (heterozygous)
Case 5	Negative	SMARCA4 c.282_283del p.(Gly95AsnfsTer33), (alias G95N*X33), AF: 8%, NM_003072.5, chr19:11,096,007, hg19 Clinically significant gene amplifications: Negative LOH of chromosome 19p: Negative TMB: Low; 4.7 mut/Mb MSI-L; 3.3% loci	Negative	Negative
Case 6*	ND	ND	ND	ND
Case 7	Negative	SMARCA4 c.2133_2134del p.(Gln712ArgfsTer4), (alias Q712R*X4), AF: 90%, NM_003072.5, chr19:11,121,065, hg19 ARID5B c.1489dup p.(Ile497AsnfsTer31), (alias I497N*X31), AF: 48%, NM_032199.3, chr10:63,850,704, hg19 CTNNB1 c.133_135del p.(Ser45del), (alias S45del), AF: 44%, NM_001904.4, chr3:41,266,133, hg19 Clinically significant gene amplifications: negative LOH of chromosome 19p: Positive (copy neutral) TMB: Low; 3.9 mut/Mb MSI-L; 0.8% loci	Negative	Negative
Case 8	Negative	*SH2B3* c.70_71del, p.(Arg24GlyfsTer7), (alias R24G*X7), AF: 27%, NM_005475.3, chr12:111,856,016, hg19 *SMARCA4* c.4024G > T, p.(Glu1342Ter), (alias E1342X), AF: 64%, NM_003072.5, chr19:11,145,662, hg19 Clinically significant gene amplifications: Negative LOH of chromosome 19p: Positive (copy neutral) TMB: Low; 5.5 mut/Mb MSS; 0% loci	Gain of chr. 14	Negative

+ in Case 6 tissue was not available for ancillary studies

*CNVs* copy number variations, *FISH* fluorescent in situ hybridization, *ND* not done, *LOH* loss of heterozygosity, *mut* mutation, *Mb* megabase, *TMB* tumor mutation burden, *MSI* microsatellite instability

#### Detailed description of cases

### SMARCA4-deficient carcinomas from non-sinonasal locations

## Case 1

A 66-year-old man with a 1-year history of odynophagia, progressive oral swelling, and bilateral cervical lymphadenopathy presented with an ulcerated exophytic tumor in the floor of the mouth (teeth 46–36), extending to the mandibular alveolar ridge, with restricted tongue mobility. He was a heavy smoker and regular alcohol consumer, otherwise healthy. Biopsy confirmed p16-negative oral squamous cell carcinoma. Clinical stage was cT4a cN2c cM0, stage IVA. The patient underwent ventral hemiglossectomy, segmental mandibulectomy [32–41], bilateral neck dissection, and reconstruction with a free fibular flap.

**Fig. 1:**
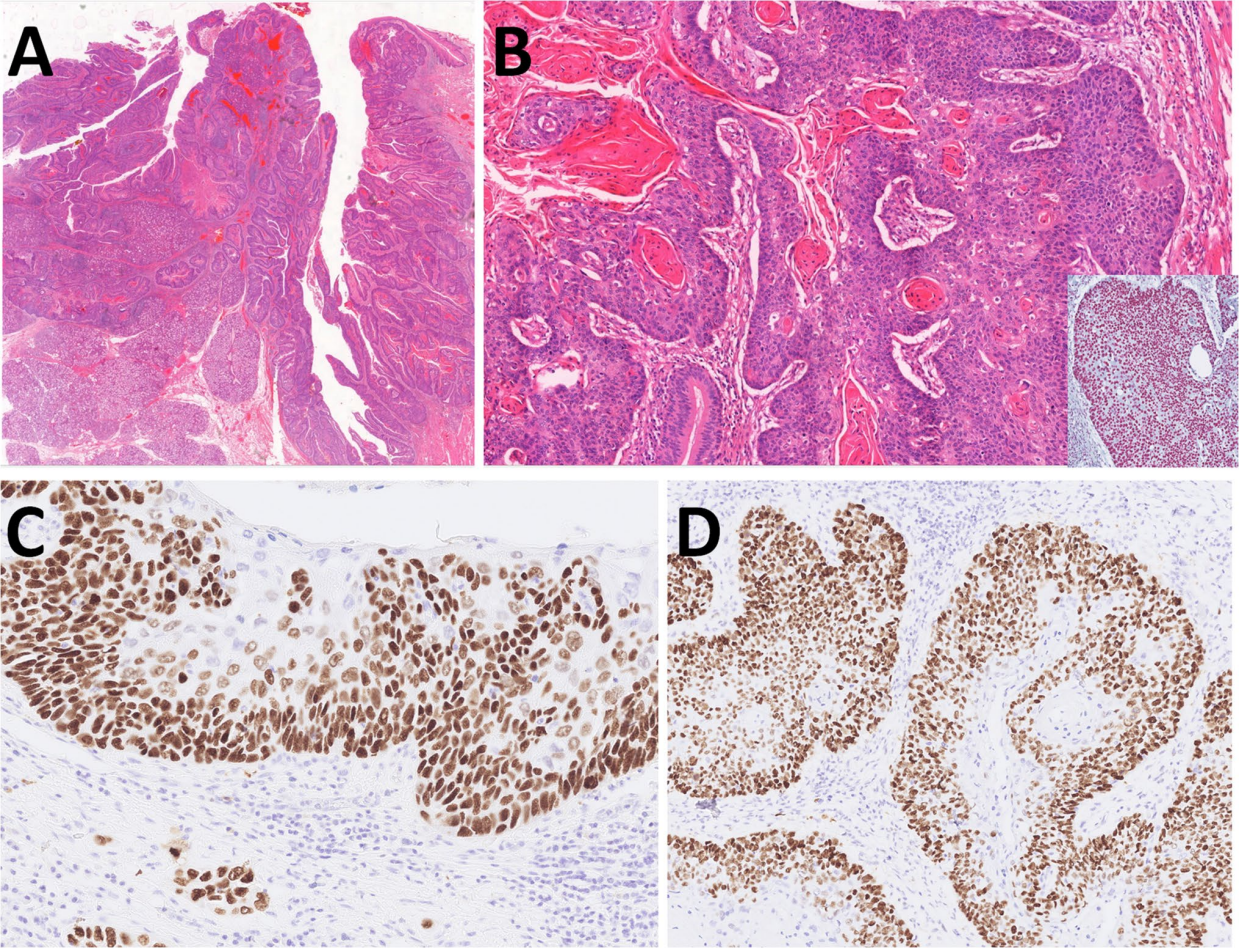
Case 1 consisted of high-grade oral epithelial dysplasia transforming into invasive well-differentiated keratinizing squamous cell carcinoma (**A**, **B**) with retained SMARCA4 expression (inset), well highlighted by p63 nuclear positivity in dysplastic areas and the invasive component. p53 showed aberrant expression (**C**)

**Fig. 2:**
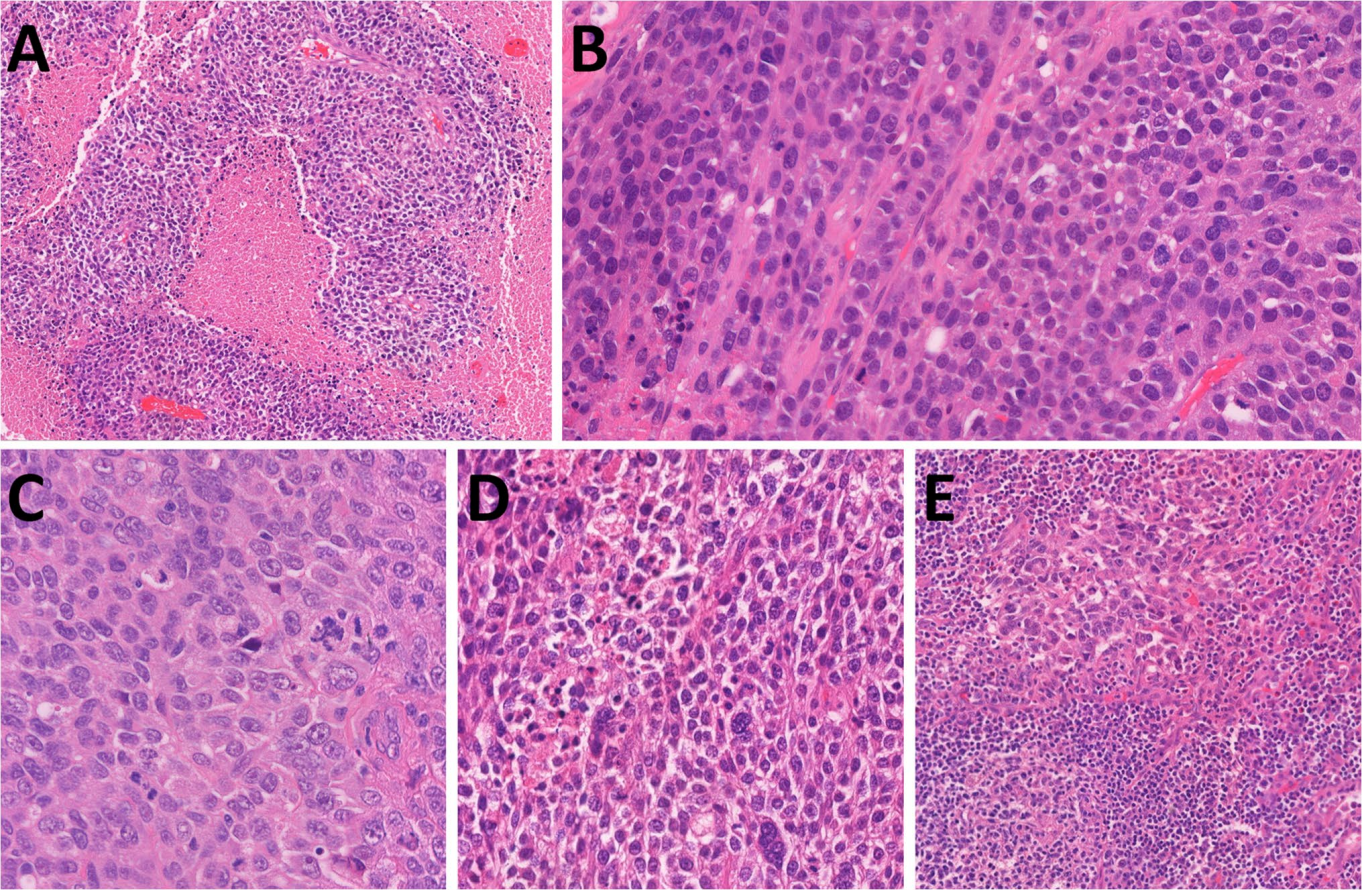
The second component of Case 1 was largely necrotic high-grade malignancy with perivascular sparing of tumor cells (**A**). The tumor was predominantly solid and, in some areas, grew in single-cell lines separated by thin fibrous stroma (**B**). It consisted of an epithelioid to rhabdoid pleomorphic tumor population with high mitotic activity, including atypical mitoses (**C**). Large and multinucleated nuclei were present throughout the tumor (**D**). Two lymph nodes were involved (**F**)

**Fig. 3:**
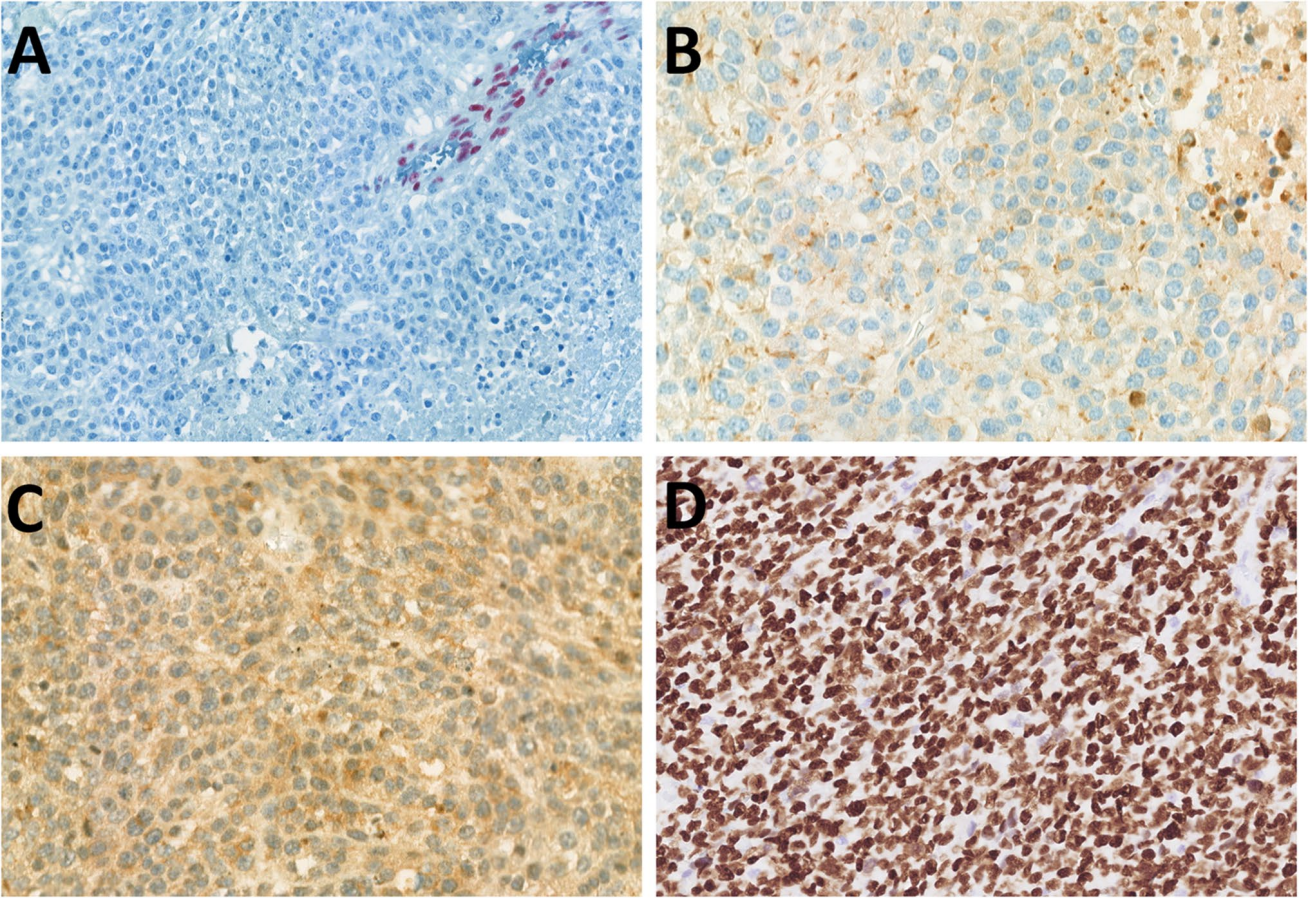
The high-grade pleomorphic component in Case 1 showed complete loss of SMARCA4 with positive inner control in endothelial cells (**A**). Cytokeratin (AE1/3) expression was weak, with a dotted cytoplasmic distribution (**B**). Synaptophysin showed diffuse but weak expression (**C**). p53 demonstrated an aberrant pattern of expression of strong nuclear positivity (**D**)

**Fig. 4:**
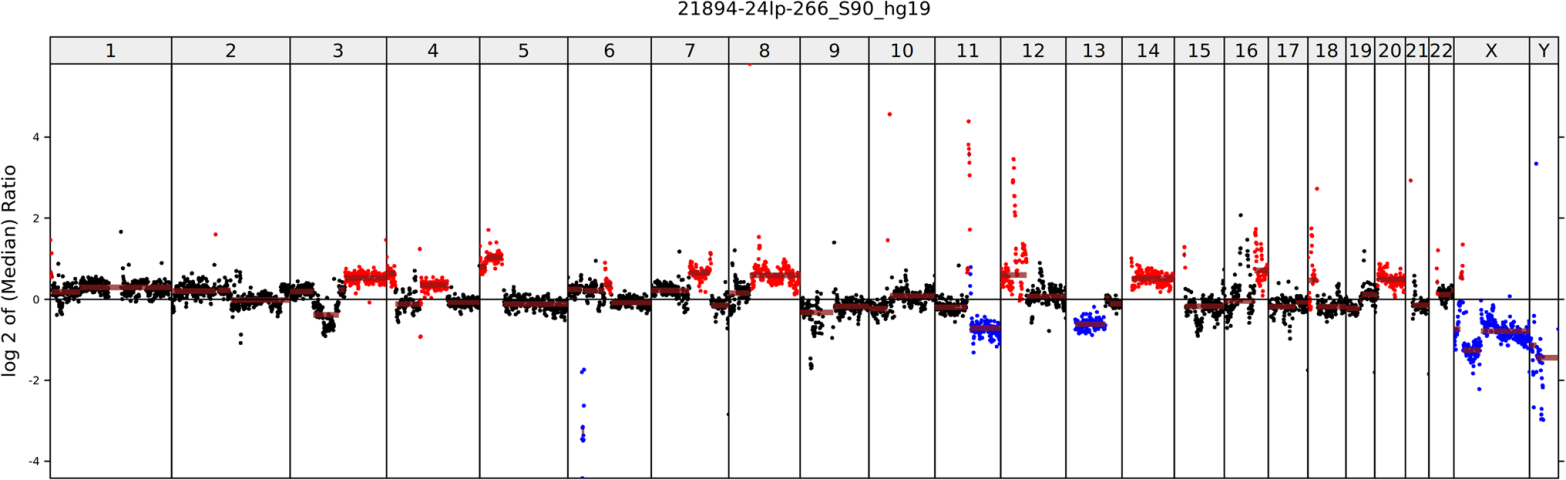
A novel fusion with unknown biological significance was detected, namely SLC66A1::PLCB1 (exon 1::exon 31) of unknown frame (NM_001040125.2, NM_015192.4, chr1:19,639,383, chr20:8,770,822, Hg19)

Gross examination of the resected specimen showed an ulcerated lesion (3 × 2.5 cm, 8 mm deep) overlying a distinct nodule (3.5 × 2.8 × 4.2 cm). Histologically, the tumor had two components. Differentiated surface component was composed of high-grade epithelial dysplasia and invasive well-differentiated keratinizing squamous cell carcinoma (SCC) (Fig. 1A, B). Immunohistochemistry showed diffuse positivity for AE1/3, CK5/6, and p63 (Fig. 1C) and negativity for CK7 and p16. P53 showed strong nuclear positivity (Fig. 1D). SMARCA4 (Fig. B, inset), SMARCA2, SMARCB1, and retinoblastoma 1 (RB1) were retained. Deeper poorly differentiated component consisted of largely necrotic tumor with perivascular sparing (Fig. 2A), solid growth with nests, single files, and occasional pseudorosettes (Fig. 2B). Tumor cells had epithelioid to rhabdoid morphology and high mitotic activity (9 mitoses/mm^2^) (Fig. 2C). Ocasionally, multinucleolated tumor cells were found (Fig. 2D). Two of 78 lymph nodes were infiltrated by this poorly differentiated component (Fig. 2E). Immunohistochemically, this component showed complete loss of expression of SMARCA4 (Fig. 3A) and partial loss of SMARCA2 (50% of cells). SMARCB1 and RB1were retained. Cytokeratin expression (AE1/3, OSCAR, MOC31, and Cam5.2) was weak, with a dotted cytoplasmic distribution (Fig. 3B). Synaptophysin was diffusely but weakly positive (Fig. 3C), other neuroendocrine markers were negative, and p53 showed strong nuclear positivity (Fig. 3D). A novel fusion, *SLC66A1::PLCB1* (exon 1::exon 31) of unknown significance, was detected by NGS (Fig. 4). In addition, a pathogenic *SMARCA4* (Gln194Ter) mutation with loss of heterozygosity (19p) was identified by NGS, and a heterozygous loss of the *SMARCA4* gene was confirmed by FISH.

In this context—where the well-differentiated SCC component showed retained SMARCA4 expression—the SMARCA4 loss in the poorly differentiated portion of the tumor likely reflects a dedifferentiation event rather than a primary driver alteration.

Final pathological staging was pT3 pN2c M0, G3, R0, and stage IVA. Adjuvant radiotherapy was deferred due to complications. At 19 months postoperatively, the patient remains in complete remission.

## Case 2

An 81-year-old man presented with a tumor involving the radix of the tongue and regional lymphadenopathy. His medical history included chronic obstructive pulmonary disease, likely smoking-related, and previously treated pulmonary sarcomatoid carcinoma (details unavailable). Incisional biopsies of the tongue lesion and a right level II lymph node were performed, followed by percutaneous endoscopic gastrostomy.

**Fig. 5:**
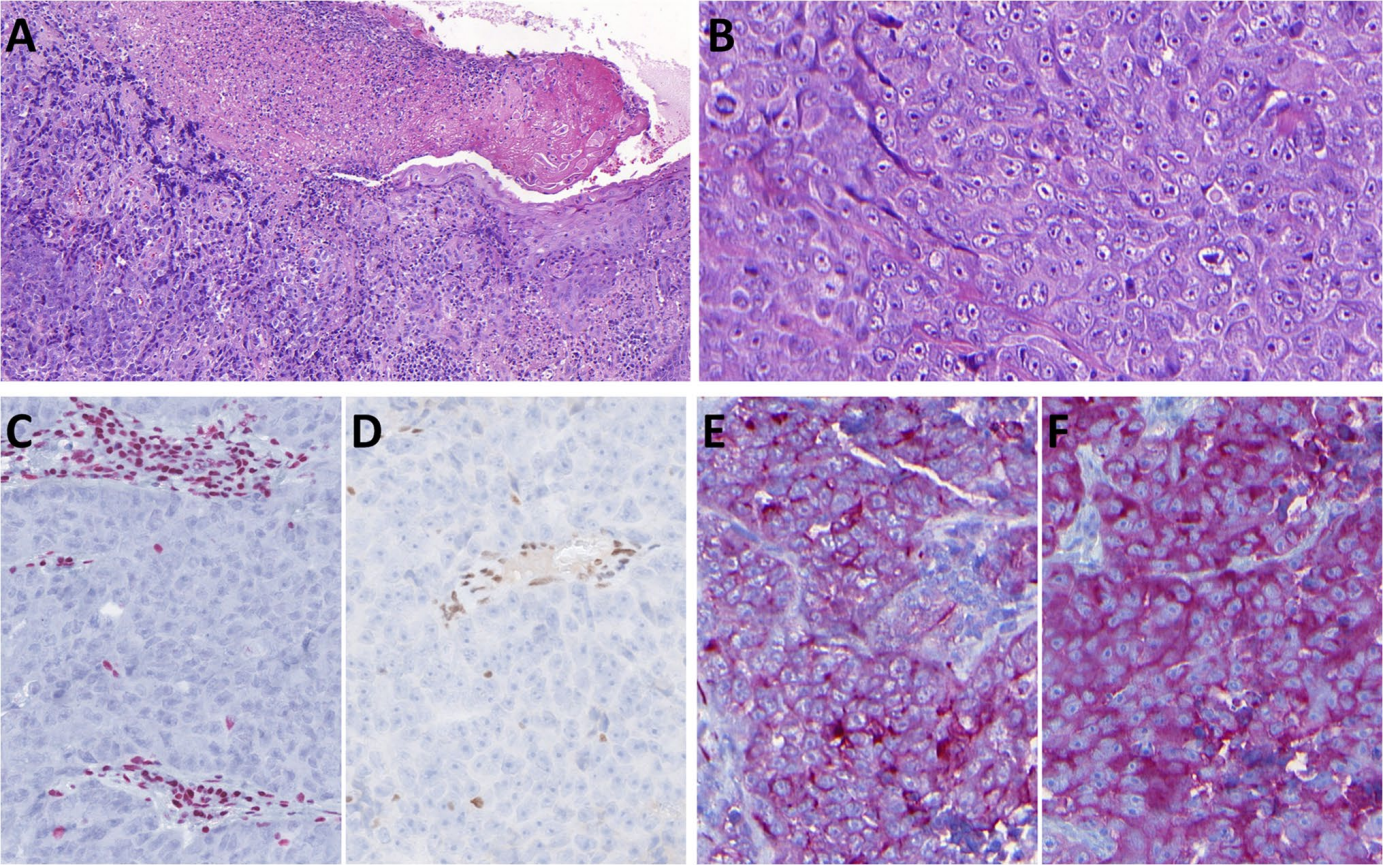
Case 2 was an ulcerated, poorly differentiated tumor (**A**) composed of a monomorphic basaloid population of tumor cells with irregular size and shape, multiple large nucleoli, and high mitotic activity (**B**). SMARCA4 antibody showed loss in tumor cells (**C**), with SMARCA2 co-loss (**D**) (both antibodies with positive internal controls in endothelial cells). AE1/3 (**E**) and synaptophysin (**F**) were positive in a diffuse membranous pattern

**Fig. 6:**
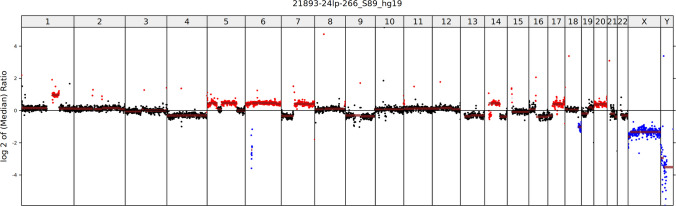
Genetically, multiple chromosomal copy number variations (CNVs) were detected

Histology revealed a poorly differentiated tumor composed of relatively monomorphic epithelioid cells with vesicular nuclei, multiple prominent nucleoli, and high mitotic activity (13 mitoses/mm^2^) **(**Fig. 5A, B). One lymph node showed metastatic involvement. Immunohistochemistry demonstrated retained expression of SMARCB1 and ARID1A, with complete loss of SMARCA4 (Fig. 5C) and SMARCA2 (Fig. 5D). Tumor cells were positive for cytokeratins (AE1/3) (Fig. 5E) and synaptophysin (Fig. 5F) and showed aberrant null-type p53 expression. Genetic analysis revealed multiple chromosomal copy number variations (CNVs), including loss of heterozygosity of chromosome 19p (*SMARCA4* locus) (Fig. 6). In addition, a novel in-frame fusion *RPS6KB1::VMP1* (exon 4::exon 6) was detected. FISH testing confirmed homozygous loss of the *SMARCA4* gene.

Due to advanced age and comorbidities, radical surgery was not pursued; palliative therapy was initiated. The patient contracted COVID-19 3 months later and died 9 days after infection. The total follow-up was three months.

## Case 3

A 61-year-old woman was referred for a second opinion regarding a tumor of the upper jaw, originally submitted as epulis and reported by the pathologist as ductal adenocarcinoma. As this is a recent case, treatment and pathological staging have not yet been conducted.

**Fig. 7:**
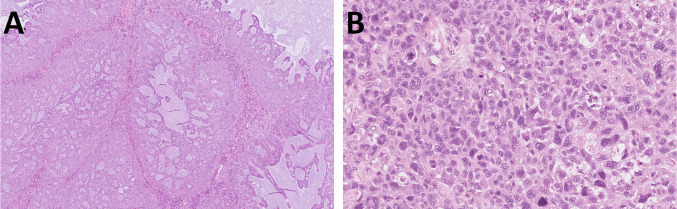
Case 3 was a necrotic tumor composed predominantly of glandular, cribriform, and ductal structures with mucin production and occasionally papillary formations with pleomorphic tumor cells and high mitotic activity (**A**). Case 4 was a pleomorphic nested to sarcomatoid malignancy infiltrating deep parts of the hypopharyngeal wall including striated muscle tissue (**B**)

Histologically, the tumor exhibited focally necrotic solid nests with glandular, cribriform, and ductal-like architecture, mucin production, and occasional pseudopapillary formations (Fig. 7A). Tumor cells were large and pleomorphic, with irregular nuclei, eosinophilic nucleoli, and high mitotic activity (10 mitoses/1 mm^2^). Immunohistochemistry showed positivity for AE1/3, CK7, and MUC4 and negativity for CK5/6, TTF1, estrogen and progesterone receptors, S100, ALK, NUT, p63, SOX10, and GATA3. SMARCB1 and ARID1A were retained, while SMARCA4 was lost. P53 expression was wild-type. Molecular analysis revealed *KRAS* mutation (*c.35G* > *C, p.Gly12Ala*). Heterozygous loss of *SMARCA4* gene was detected by FISH.

## Case 4

A 62 year old presented with an ulcerated hypopharyngeal tumor. Beneath the surface was partly necrotic, solid-growing non-keratinizing epithelioid tumor infiltrated striated muscle and showed areas of sarcomatoid transformation. The tumor showed secondary involvement of the superficial non-dysplastic epithelium, resembling carcinoma in situ. Tumor cells were large, polygonal, and plasmacytoid, with eosinophilic cytoplasm, irregular nuclei, and high mitotic activity (35 mitoses/1 mm^2^) (Fig. 7B). Immunohistochemically, the tumor cells were positive for AE1/3 and CK7 and showed retained expression of SMARCB1 and ARID1A. The tumor cells demonstrated loss of SMARCA4 expression (including in the CIS-like component), were negative for p63, and showed p40 expression in fewer than 5% of tumor cells. The patient received chemotherapy and remains alive with disease; no further follow-up is available. Heterozygous loss of the *SMARCA4* gene was detected by FISH.

### SMARCA4-deficient carcinomas from sinonasal location

**Fig. 8:**
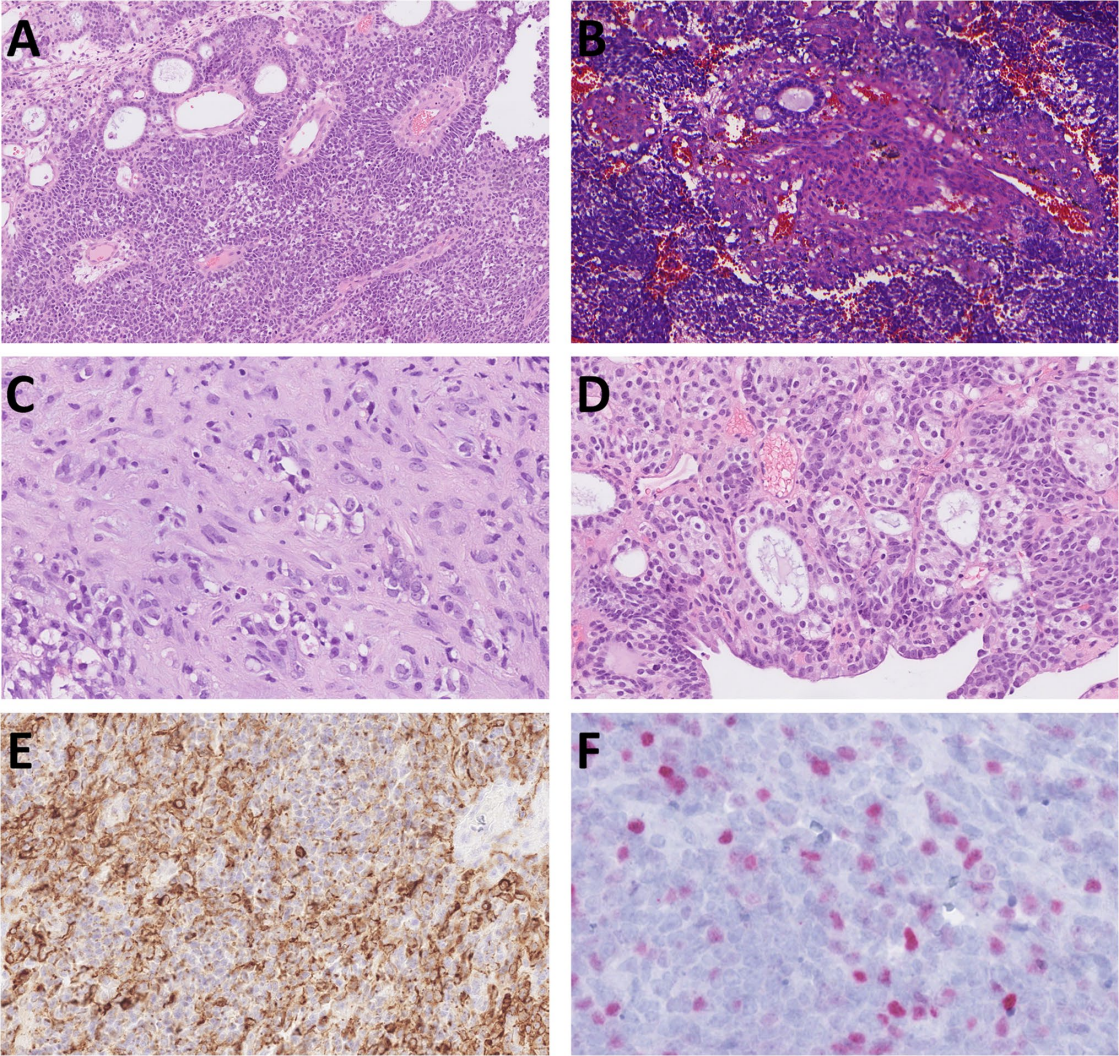
The sinonasal cases resemble ONB, presenting both true rosettes and pseudorosettes and focally with glandular architecture and predominantly small cell morphology (**A**). In Case 7, squamous areas and small glandular structures were observed within the predominantly patternless small cell morphology (**B**). In Case 5, the tumor cells were dyscohesive and pleomorphic, occasionally exhibiting spindle cell morphology (**C**). They focally displayed vacuolated cytoplasm and clustering into small groups and duct-like areas (**D**). AE1/3 was observed in most cases, at least in a focal distribution (**E**). Two cases displayed focal INSM1 expression (**F**)

Three of four sinonasal SMARCA4-deficient carcinomas (Cases 5, 6, and 8) were initially diagnosed as SMARCA4-deficient sinonasal carcinoma, while one (Case 7) was originally referred to as high-grade olfactory neuroblastoma (ONB) or neuroendocrine carcinoma (NEC). These tumors predominantly consisted of small, poorly cohesive cells arranged in lobules. In two cases, both true and pseudorosettes were present, and glandular architecture was occasionally observed (Fig. 8A). Case 7 focally showed squamous differentiation (Fig. 8B), while Case 5 exhibited dyscohesive pleomorphic cells and occasional spindle cell morphology (Fig. 8C). In Case 6, tumor cells focally displayed vacuolated cytoplasm and formed small clusters and well-differentiated duct-like structures (Fig. 8D).

Two patients were alive with disease at 0 and 24 months (treated by surgery and chemotherapy, respectively), one died at 3 months, and follow-up data were unavailable for one case. All tumors showed loss of SMARCA4 expression and at least focal positivity for cytokeratin markers (AE1/3, OSCAR, CAM5.2, and/or CK7) (Fig. 8E). Synaptophysin was positive in three tested cases, and INSM1 showed focal positivity in two (Fig. 8F).

Full genetic testing was conducted in three cases, all revealing *SMARCA4* mutations, with *ARID5B* and *CTNNB1* comutations present in Case 7 and *SH2B3* comutation in Case 8 (Table 3). Whole genome CNV analysis was negative for chromosome 19 alteration in Cases 7 and 8, while LOH of 19p was identified there by NGS, i.e., copy neutral LOH is presented in those cases. In case 5, evaluation of 19q LOH/CNV was inconclusive due to the low allele frequency of the *SMARCA4* mutation. Additionally, chromosome 14 gain was detected in case 8. All three tested cases were negative for *SMARCA4* loss by FISH.

### Review of the literature

A total of 30 cases of SMARCA4-deficient head and neck carcinomas have been reported in the English literature, including the 8 cases presented in this study. Of these, 23 patients were male and 5 were females, and sex was unspecified in 2 cases. The mean age was 52.6 years (range, 20–72 years). The sinonasal tract was the primary site of origin in 24 cases, with 6 cases arising in extra-sinonasal locations: right tonsil, floor of the oral cavity, the tongue, upper jaw, hypopharynx, and parotid gland (see Supplementary file 1).

Treatment modalities included surgery (9 cases), chemotherapy (12 cases), radiotherapy (7 cases), and immune checkpoint inhibition (ICI, 1 case, nivolumab). Two patients received no treatment, and one patient underwent biopsy only. At last follow-up, 4 patients (13%) were alive without evidence of disease (mean follow-up, 17.2 months; range, 5.6–35 months), all treated surgically, with three also receiving adjuvant radiotherapy and/or chemotherapy. Eight patients (27%) were alive with disease (mean follow-up, 9.3 months; range, 0–24 months), including three recent cases. Five of these patients received chemotherapy and one was treated by ICI. Ten patients (33%) died of disease (mean follow-up, 10.1 months; range, 1–34 months), and one (3%) died of unrelated causes with disseminated disease 3 months after diagnosis. Follow-up data were unavailable for seven cases (23%).

All cases showed loss of SMARCA4 expression by immunohistochemistry with preserved internal controls. SMARCB1 and ARID1A were retained in all tested cases (*n* = 21 and *n* = 6 cases, respectively). SMARCA2 was evaluated in 16 cases: 4 showed complete loss, 3 had heterogeneous expression, and 9 retained expression. Cytokeratin positivity was observed in all tested cases (25/25), though sometimes focal. CK7 was positive in 7 of 17 cases (41%), while CK5/6 was negative in all 15 tested cases. Synaptophysin was expressed in 19 of 23 cases (83%), Chromogranin A in 9 of 20 cases (45%), INSM1 focally in 4 of 9 cases (44%), and CD56 in 6 of 8 cases (75%). All tested cases were negative for p63, TTF1, and NUT (see Table 2).

## Discussion

The SWI/SNF complex is a multi-subunit, ATP-dependent chromatin-remodeling complex involved in transcriptional regulation and cell differentiation. Mutations in its components are implicated in approximately 20% of human cancers [42]. The complex consists of 12–15 subunits encoded by 29 genes, which collectively contribute to transcriptional regulation across various cell types and developmental stages [3, 42–45]. Modulations of specific SWI/SNF subunits at particular stages of cell determination govern the final cell phenotype, ranging from completely undifferentiated/anaplastic to site-specific morphology [32, 33, 46].

Advances in immunohistochemistry and molecular genetics, including methylation profiling [34], have led to the reclassification of sinonasal undifferentiated carcinoma (SNUC) into genetically defined subtypes of highly aggressive malignancies [35], including NUT carcinoma [36] and SWI/SNF-deficient sinonasal cancers, which comprise SMARCB1-deficient sinonasal carcinoma [37, 38], SMARCB1-deficient adenocarcinoma [39–41], SMARCA4-deficient sinonasal carcinoma [23, 24], and SMARCA4-deficient sinonasal teratocarcinosarcoma [21]. Unlike genuine SNUCs, SWI/SNF-deficient sinonasal neoplasms lack *IDH2* mutations [35, 47–49]. Interestingly, a multi-omics approach has identified four molecular subgroups of SNUCs: NEC-like IDH2 mutant, SMARCB1-deficient, adenoid cystic carcinoma-like, and NEC-like SMARCA4/ARID1A-deficient [34]. The later class consisted of tumors with broad differential diagnoses including SNUC, NEC, ONB, adenocarcinoma, poorly differentiated carcinoma, and squamous cell carcinoma. Histologically, rosette-like architecture and at least weak neuroendocrine markers expression were common. These tumors may occur in the context of rhabdoid tumor predisposition syndrome 2. Activating mutations in *PIK3CA* (32%) and other oncogenes (*CTNNB1*, *TP53*, *TSC2*) have been reported, suggesting potential therapeutic targets [37]. In contrast, PI3K-pathway alterations are rare in IDH-mutant SNUCs [48, 49]. While response to PI3K-pathway inhibition remains uncertain, based on preliminary data, immune checkpoint inhibitors have shown promise in SMARCA4/ARID1A deficient thoracic tumors [50, 51].

It was suggested that the prognosis for NEC-like SMARCA4/ARID1A class may be quite favorable, with reported 5-year survival rates of 68%, comparable to IDH2-mutant NEC-like tumors (59%) [34]. In the present study, 5 patients (63%) were alive without or with the disease after a mean follow-up period of 8 months (range, 0–24 months).

Histologically, head and neck SMARCA4-deficient carcinomas are often misdiagnosed as small or large cell NEC or ONB [23, 24]. Most sinonasal cases resemble SCCOHT or thoracic SMARCA4-deficient carcinomas, featuring basaloid or large epithelioid cells with abundant cytoplasm, prominent nucleoli, high mitotic activity, and necrosis. Architectural patterns include nests, lobules, and trabeculae, mimicking IDH-mutant SNUCs. Rhabdoid and small cell features are less common than in SMARCB1-deficient tumors [23, 24, 52]. In our series, one case (Case 1) showed a clear sequence of dysplasia—well differentiated SCC—dedifferentiated SMARCA4-deficient carcinoma. The surface epithelium was evaluable in only one additional case (Case 4), which demonstrated SMARCA4-deficient tumor cells secondarily involving the superficial, non-dysplastic epithelium, thus mimicking carcinoma in situ. Given the limited number of cases, no conclusions can be drawn about the type of differentiation in extra-sinonasal tumors.

Immunohistochemically, these tumors show variable pancytokeratin expression and less frequent CK7 positivity [23]. Claudin-4 appears to be a useful surrogate marker for distinguishing SWI/SNF complex-deficient carcinomas from sarcomas, showing positivity in 80% of tested cases, compared to only 4% of sarcomas with epithelioid morphology (excluding synovial sarcoma) [53]. In contrast to these published data, Claudin-4 expression in our cohort was lower: only 2 of 5 tested cases (40%) showed positivity, while 3 of 5 cases were negative. This discrepancy may reflect the limited cohort size, tumor heterogeneity, or technical and interpretative differences in immunohistochemical assessment. Neuroendocrine markers such as synaptophysin and chromogranin are variably expressed, while INSM1 is often negative or focal, aiding differentiation from NEC. Classical NEC features like stippled chromatin and nuclear morphology are typically absent [24, 54]. Some cases, however, show immunophenotype overlapping with NEC, with Rb1 loss and p53 overexpression [24].

Head and neck SMARCA4-deficient carcinomas can be differentiated from NUT-carcinoma using squamous markers, NUT immunostaining, or *NUT* rearrangement [55]. High-grade ONB lacks significant cytokeratin expression and typically displays lobular architecture with rosettes, along with the expression of neuroendocrine markers, calretinin, and SSTR2A [56–58]. Notably, as demonstrated in this study, some SMARCA4-deficient sinonasal carcinomas may also exhibit rosettes or pseudorosettes and commonly express neuroendocrine markers, which can be misleading in the absence of SMARCA4 immunostaining. Finally, an emerging entity, sinonasal tumor with neuroepithelial differentiation (also referred to as olfactory carcinoma), must be excluded. These tumors are diffusely positive for pancytokeratins and neuroendocrine markers, frequently contain complex and often ciliated glands, and have a variable presence of S100-positive sustentacular cells [59].

In thoracic SMARCA4-deficient undifferentiated tumors, a strong association with heavy smoking exposure and high tumor mutational burden (TMB) has been consistently reported [60]. However, the applicability of this paradigm to SMARCA4-deficient tumors arising in the head and neck region remains uncertain. In the present cohort, the majority of tested cases demonstrated low TMB and microsatellite-stable or MSI-low status, despite their clinically aggressive behavior. These findings suggest that head and neck SMARCA4-deficient tumors may constitute a biologically distinct subgroup, in which tumor progression is primarily driven by epigenetic dysregulation and chromatin remodeling defects associated with SMARCA4 loss, rather than by accumulation of somatic mutations. Notably, although a history of smoking was present in selected cases, smoking exposure did not uniformly translate into a high TMB in this non-thoracic context. Together, these observations indicate that the smoking-associated, TMB-high model described in thoracic SMARCA4-deficient tumors cannot be directly extrapolated to H&N counterparts, which appear to follow a different mutational and biological trajectory.

Treatment remains challenging for SMARCA4-deficient malignancies due to the rarity and aggressive nature of these tumors. Current strategies are largely extrapolated from other high-grade cancers and include surgery, adjuvant or neoadjuvant chemotherapy, and/or radiotherapy. Recently, the therapeutic landscape has expanded to include immune checkpoint inhibitors (pembrolizumab, nivolumab, atezolizumab), EZH2 inhibitors (tazemetostat), synthetic lethality approaches (e.g., ataxia telangiectasia and Rad3-related protein inhibitors—ceralasertib and berzosertib—and PARP inhibitors—olaparib), and bromodomain and extraterminal domain (BET) inhibitors [3, 61, 62]. Multimodal therapy has the potential to improve outcomes, and clinical trials are emerging for optimizing care.

## Conclusion

SMARCA4-deficient carcinomas of the head and neck are highly aggressive and are often diagnosed at an advanced stage. Approximately one-third of patients succumb to the disease within the first 10 months of follow-up. Their marked morphological heterogeneity poses a diagnostic challenge, as they can mimic various undifferentiated or poorly differentiated malignancies, including olfactory neuroblastoma, neuroendocrine carcinoma, and others. Given their broad differential diagnosis, accurate classification requires comprehensive immunohistochemical and molecular analysis, including SMARCA4 and SMARCB1 status. This is essential for guiding appropriate patient management and exploring emerging therapeutic options. Finally, we have detected two gene fusions of unknown significance including *SLC66A1::PLCB1* and *RPS6KB1::VMP1*.

## Supplementary Information

Below is the link to the electronic supplementary material.

ESM1 (DOCX 22.3 KB)

## Data Availability

All data generated or analyzed during this study are included in this published article [and its supplementary information files]. The complete datasets generated during and/or analyzed during the current study are available from the corresponding author upon reasonable request.
