# Supplementary material for: SMARCA4-deficient carcinoma of the head and neck region: report of 8 new sinonasal and non-sinonasal cases and literature review
Source: Virchows Arch. 2026 Mar 9;488(5):1021–37. doi: 10.1007/s00428-026-04459-5 (PMC13176017; doi:10.1007/s00428-026-04459-5)
Supplement: Supplementary file 1 — (DOCX 22.3 KB) [file 428_2026_4459_MOESM1_ESM.docx]

**Supplementary file 1. Literature review of SMARCA4 deficient H&N carcinomas**

| **Study** | **Case** | **Age/ Sex** | **Site** | **Initial diagnosis** | **Therapy** | **FU status** | **FU (mo)** | **Stage** | **PMID** |
| --- | --- | --- | --- | --- | --- | --- | --- | --- | --- |
| **Current study Bradová et al.** | **Case 1** | 66/M | oral cavity floor | collision tumors squamous cell carcinoma and SMARCA4-defficient carcinoma | Surgery | ANED | 19 | T3N2c M0, G3, R0, stage IVA | none |
|  | **Case 2** | 81/M | tongue and cervical lymph nodes | SMARCA4/BRG1-deficient carcinoma | none | DOUR | 3 | NA | none |
|  | **Case 3** | 61/F | Upper jaw | ductal adenocarcinoma | none | recent case AWD | 0 | NA | none |
|  | **Case 4** | 62/M | Hypopharynx | SMARCA4/BRG1-deficient carcinoma | CHT | recent case AWD | 0 | NA | none |
|  | **Case 5** | NA/NA | Sinonasal | SMARCA4/BRG1-deficient carcinoma | NA | NA | NA | NA | none |
|  | **Case 6** | 37/M | Sinonasal | SMARCA4/BRG1-deficient carcinoma | CHT | AWD | 24 | NA | 36206446^#^ |
|  | **Case 7** | 67/M | Sinonasal | High grade ONB or NEC | Surgery | DOD | 3 | cT4bcN0 | none |
|  | **Case 8** | 68/M | Sinonasal | neuroendocrine SMARCA4/BRG1-deficient  carcinoma | CHT | recent case AWD | 7 | T4aN0M0 | none |
| **2017 Jo, et al.** | Case 8 | NA/NA | Sinonasal | NA | NA | NA | NA | NA | 28084339 |
| **2020 Agaimy, et al.** | Case 1 | 40/F | Sinonasal | NEC | Surgery, RT | ANED | 9 | T4N2M0 | 28176137, 31934917 |
|  | Case 2 | 50/M | Sinonasal | NEC vs ONB | NA | NA | NA | NA | 31934917 |
|  | Case 3 | 20/M | Sinonasal | Large cell NEC | CHT | DOD | 3 | T4NXM1 | 31934917 |
|  | Case 4 | 47/M | Sinonasal | Large cell NEC | CHT | AWD | 8 | T4NXM1 | 31934917 |
|  | Case 5 | 30/M | Sinonasal | NEC | NA | NA | NA | NA | 31934917 |
|  | Case 6 | 41/M | Sinonasal | Poorly differentiated malignant tumor | NA | NA | NA | NA | 31934917 |
|  | Case 7 | 51/F | Sinonasal | Small cell NEC | Surgery | DOD | 3 | NA | 31934917 |
|  | Case 8 | 42/F | Sinonasal | Poorly differentiated carcinoma | CHT | DOD | 7 | T4NXM1 | 31934917 |
|  | Case 9 | 67/M | Sinonasal | Poorly differentiated carcinoma | NA | NA | NA | T4NXMx | 31934917 |
|  | Case 10 | 54/M | Sinonasal | SNUC | Biopsy only | DOD | 1 | T4NXMx | 31934917 |
| **2021 Kakkar, et al.*** | Case 1 | 48/M | Sinonasal | Poorly differentiated NEC, small cell type | CHT, excision, RT | ANED | 35 | NA | 34871352 |
|  | Case 2 | 70/M | Sinonasal | Poorly differentiated NEC, large cell type | CHT, excision, RT | DOD | 9 | NA | 34871352 |
|  | Case 6 | 30/M | Sinonasal | High-grade poorly differentiated carcinoma with focal neuroendocrine differentiation | CHT | DOD | 34 | NA | 34871352 |
|  | Case 7 | 43/M | Sinonasal | Poorly differentiated NEC, small cell type with overlaying squamous dysplasia | None | DOD | 34 | NA | 34871352 |
| **2021 Mibayashi, et al.** | Case | 50/F | Sinonasal | ONB | immune checkpoint inhibitor (nivolumab) | AWD | 12 | cT4bN2cM0 | 33881196 |
| **2023 Pasricha, et al.** | Case | 67/M | Right tonsil | SMARCA4/BRG1-deficient carcinoma | CHT, RT | DOD | 1 | NA | 38457069 |
| **2024 Gyu Kang, et al.** | Case 1 | 72/M | Sinonasal | ONB/Sinonasal carcinoma | CHT, RT | AWD | 17.5 | pT4bNxM0 | 38351682 |
|  | Case 2 | 50/M | Sinonasal | ONB/Sinonasal carcinoma | surgery, RT | NED | 5.6 | pTxN2bM0 | 38351682 |
| **2024 Zhu et al.** | Case 1 | 34/M | Sinonasal | SMARCA4/BRG1-deficient carcinoma | CHT, RT | DOD | 6 | NA | 38989233 |
|  | Case 2 | 66/M | Sinonasal | SMARCA4/BRG1-deficient carcinoma | surgery, CHT | AWD | 6 | NA | 38989233 |
| **2024 Bal, et al.** | Case | 60/M | Parotid | SMARCA4/BRG1-deficient carcinoma ex pleomorphic adenoma | surgery | NA | NA | NA | 39502002 |

* cases 3-5 were previously reported by Agaimy et all PMID:31934917

^#^This case was included in the study by Rooper et al. discussing teratocarcinosarcomas; however, morphologically, this tumor does not contain primitive neuroepithelial elements nor a mesenchymal component. Therefore, we concluded that the correct diagnosis is SMARCA4-deficient carcinoma, not teratocarcinosarcoma (MB, AA, AS, and MŠ). This case was also presented at the 34th International Congress of the International Academy of Pathology (IAP) in Sydney, held from October 11–12, 2022.

ANED – alive not evidence of disease, AWD – alive with disease; CHT – chemotherapy; DOD – died on disease; DOUR – died of unrelated reasons; FU – follow-up; mo – months; NA – not available; NEC – neuroendocrine carcinoma; ONB – olfactory neuroblastoma; RT – radiotherapy
